# Twelve tips for peer teaching by a medical education student society

**DOI:** 10.12688/mep.19928.2

**Published:** 2024-05-17

**Authors:** Sowmya Prasanna Kumar Menon, Balamrit Singh Sokhal, Vittal Rao, Fidelma O'Mahony, Janet Lefroy

**Affiliations:** 1 School of Medicine, Keele University, Keele, England, ST5 5BG, UK; 2 University Hospitals North Midlands, Stoke-on-Trent, UK

**Keywords:** medical education, teamwork, medical students, peer teaching, medical school

## Abstract

University societies are student-led organisations which provide excellent opportunities for students to collaborate in a shared interest. Peer teaching is gaining recognition as an effective method of medical education. Peer teaching also provides student tutors with core educational skills and provides students with approachable peer mentors. This article offers practical guidance on organising, planning, executing and sustaining peer teaching via a medical education society at university and outlines the supporting literature.

## Introduction

Medical schools aim to ensure that students acquire the knowledge and skills required for clinical training. This entails students spending time on clinical placements with qualified healthcare professions. Doctors must therefore be prepared not only to educate patients but students and peers. Doctors are indeed required by the General Medical Council to develop as an educator ( GMC advises doctors as teachers, 1999). Teaching is desirable for post-graduate career progression, consolidating learning and to develop leadership and mentorship skills ( Jefferis, 2007). Despite teaching being universally accepted across all medical training programmes, physicians often receive very little formal training ( Spencer, 2003). Therefore, teaching is an area of clinical activity that must be developed and supported to sustain and enrich medical education and ensure safe and professional practice amongst health care professionals. The skills of teaching can and arguably should be introduced early and practised regularly in medical school. One method to develop the clinician as an educator is through peer-teaching practices.

Peer teaching has been shown to be comparable to faculty-led teaching in terms of knowledge and skills outcomes ( Rees *et al*., 2016). It contributes positively to the personal and professional development of students and increases self-confidence and fulfilment ( Yang *et al*., 2022). Despite its benefits, peer teaching has been an underutilised initiative in medical school, therefore structured ways to implement it should be encouraged and explored ( Ten Cate & Durning, 2007). One way to do this is through the creation of a university medical education society. Societies are student-led organisations at universities that allow students with a shared interest to collaborate and engage in enriching activities. This article outlines practical and conceptual tips to develop, implement and expand a medical education society focussed on peer-teaching practices.

## Tip 1. Find the gap

The current medical curriculum is broad and comprehensive. Therefore, to maximise the educational impact of the society, it is important to focus on areas in the curriculum which might benefit from supplementation by peer teaching. These can be identified from the perspective of students through surveys and focus groups. An effective digital survey is low resource, efficient, easy to complete and accessible through the distribution of QR codes ( Nikiforova *et al*., 2021). The survey should gather information about the year of the student and desired areas for further teaching to provide a broad representation of the student views. The number of respondents allows for a rough indicator of interest. Focus groups provide qualitative and granular information. In-depth focus groups encourage students to reflect on weak areas of learning, discuss where there is disagreement, and explore potential solutions to problems ( Stalmeijer *et al*., 2014). Surveys and focus groups are invaluable to planning potential content. Senior students, the medical curriculum and lecturers should also be consulted to identify gaps in knowledge and skills based on their experience of assessments and clinical practice.

## Tip 2. Develop a content plan

Answers to surveys can be organised by topics and sub-topics. Depending on the format of the medical school’s curriculum, the overarching topic can be organised by system (e.g. cardiovascular), speciality (e.g. cardiology) or presentation (e.g. chest pain), and therefore, the society should aim to be flexible in its approach and tailor to the university and student preference. Once relevant topics have been identified, they can be divided into sub-topics for which 15–25 minute ‘stations’ each representing an aspect to be covered. For example, if the session topic is chest pain, stations for aspects to cover might be acute myocardial infarction, electrocardiogram interpretation, pneumonia etc. With a list of prospective topics to be covered, a content plan can be created which outlines the topic, date, time and duration of each session and title of each station with the intended learning outcomes. The learning outcomes should then be shared with the peer tutors to ensure that high-quality teaching of the relevant topics is provided.

## Tip 3. Devise the format

Since topics will have been introduced in the learners’ curriculum, sessions should aim to cover high-yield topics in a revision format, as a supplement to lectures delivered by faculty. The attention span of students is commonly referred to as lasting between 15–20 minutes, although this is debated ( Bradbury, 2016). As a guide, subtopic stations should therefore last around 15–25 minutes each and have short breaks in between, with the whole session lasting no longer than 2 hours. This allows the core information to be delivered with a summary of the topic at the end. Throughout the station, the tutors should encourage active problem-solving and critical thinking by asking questions. Ideally, students should be able to test their knowledge at the end through a short quiz. This will allow them to identify further areas of improvement.

## Tip 4. Teach the teacher

A training session should be held with potential senior student tutors to outline key principles of teaching. Including effective methods of teaching as agreed by the society (e.g. active recall, case based discussions etc), how to reference, how to avoid plagiarism and how to ensure learning objectives are met. This means a baseline standard for which teaching is delivered is established as a means of quality assurance and build key academic skills amongst tutors. These sessions should be developed in collaboration with faculty lecturers to incorporate their extensive experience and advice.

## Tip 5. Host on an accessible and free platform

Sessions can be done in-person or virtually. There are strengths and limitations to both which are beyond the scope of this article. Briefly, strengths of virtual sessions include accessibility, ability to record sessions for playback and low resource use. Limitations include restriction of ability to teach practical skills, difficulty ascertaining visual cues from students and lack of attentiveness from students’ resources are distributed ( Mukhtar *et al*., 2020). Strengths of in-person sessions include visual and verbal discussion whilst there limitations are due to travel, need to book rooms and less sustainable resources ( Kumari *et al*., 2021). For theory best sessions, an online approach is optimal as there is less need to have practical resources. On the other hand, practical based sessions (e.g. Objective Structure Clinical Examinations (OSCE) and anatomy) would benefit from an in-person approach where models and non-verbal skills can be demonstrated, and students are actively encouraged to participate directly through the use of spotter questions or mock OSCE. When choosing a virtual platform, the platform must be accessible, free and simple to use. Platforms for healthcare related teaching such as MedAll are free and easily accessible as long as there is an internet connection. Recordings, feedback forms and certificates can be automatically distributed, and attendance can be monitored. These methods facilitate easier evaluation of the quality of teaching and simple implementation of improvements.

## Tip 6. Create accompanying resources

An output from sessions should be resources. This includes the resources directly related to the session (e.g. presentation slides, case discussed, answers to exam questions etc), and resources produced to supplement the session. Comprehensive and accessible resources empower peer tutors to engage in different teaching methodologies and allow another form of interaction with students. Resources allow students to retrospectively view the session and aid in recalling the contents to supplement their revision. This could be a further set of questions, visual aids, condensed notes etc. at the tutor’s discretion. All resources should be checked by an independent person, for instance the session lead or society peer-reviewers to ensure the session learning objectives are being met, the resources are high quality, and the work is original and correctly cited.

## Tip 7. Advertise and leverage social media

Leveraging social media platforms to promote upcoming events helps to maximize attendance and fosters a sense of community. There are almost 4 billion social media user worldwide. Social media can improve communication between medical educators (at all stages of their career) and flatten hierarchies, contributing to wider outreach ( Guckian *et al*., 2021). Events must be advertised early and proactively re-advertised to allow the target audience to prepare in advance at their discretion, for instance by reviewing faculty-taught material and identifying areas they want further clarification on. Furthermore, this increases the chance of attracting a larger and more diverse audience. Platforms like Facebook, Twitter, and Instagram enable societies to share event details, including date, time and locations of sessions to followers, who can in-turn share the same details, potentially leading to snowballing of audience.

## Tip 8. Feedback, feedback, feedback

Feedback underpins the success of the society and is of paramount importance. Feedback should seek to evaluate the performance of the teaching quantitatively and qualitatively against the learning objectives of the session and the learning needs of the student. This should be done for each station and the overall session. Feedback has several benefits. Feedback allows the responder to reflect on their teaching qualitatively, simultaneously outlining to the society what worked well and what could have been better for the student. Feedback allows all stakeholders (teacher, society and student) to determine if the session improved quantitative performance (recommended by a pre and post station quiz for all stations). Feedback allows the tutor to improve their teaching methods where students highlight specific aspects. With common areas of improvement being clearer slides, more concise explanations or more time on a specific area. Finally, feedback allows the society to implement changes to grow and strengthen the quality of teaching ( Tuma & Nassar, 2023). For granular analysis of feedback, qualitative answers can be compared to quantitative performance to determine trends associated with an adopted teaching style. For instance, stations centred around cases lead to positive qualitative feedback and high post session quiz scores. Artificial intelligence (AI) tools could also be used to enhance the efficiency of the feedback process and present data comprehensively. However, these tools must be used with caution as we are still in the early stages of implementation of AI.

## Tip 9. Recognise the team

Peer tutors should be provided with acknowledged for their teaching contribution. Certificates for peer tutors for their engagement in pedagogical activities recognises their achievement and acts as evidence of teaching experience, thereby contributing to academic portfolios and ability to secure future roles in education. Certificates also promote the value of peer teaching and encourage more students to actively engage in the approach, fostering a supportive learning environment. Commonly training programme portfolio criteria state that for evidence of teaching to be acknowledged, evidence must be accompanied with a letter which confirms involvement in the teaching programme and the dates of the teaching, signed by a consultant with the consultant’s name and national medical registration number ([Internet] NHS Core Surgical Training Portfolio Requirements, 2022; [cited 2023 Jul 24]. Available from: https://medical.hee.nhs.uk/medical-training-recruitment/medical-specialty-training/surgery/core-surgery/core-surgical-training-self-assessment-scoring-guidance-for-candidates). Therefore the society should seek to officially validate certificates by liaising with a staff member that can facilitate this (e.g. medical school or hospital dean).

## Tip 10. Develop and improve

Feedback is only useful if acted upon in accordance with the plan-do-study-act cycle (PDSA). Feedback should be gathered immediately after each session and incorporated at the earliest convenience when planning the next session. Attendees are heterogenous as each have their own way of processing and learning interventions. There will be broad areas to improve which will increase quality of sessions for the majority of students. Areas that commonly require attention are the depth of content covered, station times, session times and the method of teaching. Listening to feedback fosters trust between attendees and the society facilitating motivated and granular feedback.

## Tip 11. Be active

Multiple sessions should be planned in advance and function in parallel with the curriculum in order to supplement faculty taught sessions. As these sessions will be organised outside of curriculum hours, they will supplement that core teaching that has been provided. Staging regular sessions allows continuity between sessions and a relationship between attendees and the society, facilitates easier re-evaluation of interventions to improve sessions, and allows growth of the society.

## Tip 12. Collaborate and broaden scope

Collaboration is key to a thriving student society. Network with other universities, students and faculty to share events, ideas, experiences and promote a rich and better quality of education. As relationships develop, it becomes easier to recruit for teaching and expand the scope of the society by providing sessions other than teaching such as mock knowledge examinations, mock OSCEs and special guest talks. This helps promote the society, empowers students and develops a culture of sustained learning within the community. This can be extended to the wider health professions community. Interprofessional collaboration in sessions such as mock OSCEs through simulation can provide invaluable insight into other healthcare roles such as pharmacy, nursing and physiotherapy.

## Conclusions

A medical education student society underpinned by peer teaching practices is a fantastic initiative to foster a medical education student community benefitting both peer tutors and learners. Senior medical student engagement in peer teaching provides them with an excellent opportunity to develop several key teaching skills. Peer tutors learn valuable pedagogy skills and methods. They gain practical experience fundamental for when they will be responsible for teaching peers and students in clinical practice. Peer tutors learn to gather feedback and implement change in their practice to improve their teaching and learn key academic skills such as referencing to deliver their teaching. They gain skills in leadership when leading sessions. Finally, peer tutors develop key relationships as mentors to the students they teach, building a sense of community and a rich learning environment.

For student learners, they gain access to resources to supplement faculty learning in areas where the medical curriculum is felt to need strengthening. Students have positive interactions with peers in the years above, especially with tutors who teach multiple sessions. Peer tutors have already been through the medical school experience of the student learners who are therefore able to obtain tailored advice, feel more comfortable asking questions and most importantly, have multiple mentors invested in their education throughout their journey. Students also get added opportunities to consolidate their learning in an active manner in addition to testing their retention. Importantly, students may well feel inspired to engage in teaching at an early stage in their career which enormous potential benefits later in their career when they are educators in clinical practice.

Medical education student societies’ peer education activities can be successfully implemented in medical schools in a variety of settings such as online or in-person and for a variety of content such as theory, practical examinations and clinical skills. The practical tips summarised in this article outlines the aspects of building the foundations of a successful medical education society.

## Data Availability

No data are associated with this article.
