# Supplementary material for: Twelve tips for peer teaching by a medical education student society
Source: MedEdPublish (2016). 2024 May 17;13:294. Originally published 2023 Nov 29. [Version 2] doi: 10.12688/mep.19928.2 (PMC11344874; doi:10.12688/mep.19928.2)
Supplement: Peer review history

# Reviewer report: Reviewer response for version 2

**DOI:** 10.21956/mep.21866.r37035

**Published:** 2024-07-05

**Authors:** Zakia Dimassi

**Affiliations:** 1 Khalifa University, Abu Dhabi, United Arab Emirates

After revisiting the manuscript, I have come to the conclusion that it still lacks sufficient substance to be indexed as an article. I appreciate the effort the authors have made to incorporate the peer-review suggestions, however, the manuscript still falls short in terms of practical application examples, integration into a curriculum, and alignment with other teaching methods. Therefore, I must recommend the article as not approved. Thank you for the opportunity to review this work.

If evidence from practice is presented, are all the underlying source data available to ensure full reproducibility?

Not applicable

Is the topic of the practical tips discussed accurately in the context of the current literature

Partly

Are all factual statements correct and adequately supported by citations?

Partly

Are the conclusions drawn balanced and justified on the basis of the presented arguments?

Partly

Are arguments sufficiently supported by evidence from the published literature and/or the authors’ practice?

Partly

Reviewer Expertise:

curriculum development and evaluation, international medical graduates, team based learning, simulation-based education, patient safety in the medical curriculum

I confirm that I have read this submission and believe that I have an appropriate level of expertise to state that I do not consider it to be of an acceptable scientific standard, for reasons outlined above.

# Reviewer report: Reviewer response for version 2

**DOI:** 10.21956/mep.21866.r37036

**Published:** 2024-06-18

**Authors:** Masego B. Kebaetse

**Affiliations:** 1 University of Botswana, Gaborone, Botswana

While the authors have improved the manuscript, the main outstanding issue is that the tips are generally barely supported or not supported with the literature.

If evidence from practice is presented, are all the underlying source data available to ensure full reproducibility?

Partly

Is the topic of the practical tips discussed accurately in the context of the current literature

Partly

Are all factual statements correct and adequately supported by citations?

Partly

Are the conclusions drawn balanced and justified on the basis of the presented arguments?

Yes

Are arguments sufficiently supported by evidence from the published literature and/or the authors’ practice?

No

Reviewer Expertise:

instructional design, teacher professional development, learning development/support

I confirm that I have read this submission and believe that I have an appropriate level of expertise to confirm that it is of an acceptable scientific standard, however I have significant reservations, as outlined above.

# Reviewer report: Reviewer response for version 2

**DOI:** 10.21956/mep.21866.r37037

**Published:** 2024-06-07

**Authors:** Prattama Santoso Utomo

**Affiliations:** 1 Universitas Gadjah Mada, Yogyakarta, Indonesia

The authors have addressed most of my previous suggestions and revised the manuscript. Overall the quality of the manuscript has been improved, despite of a citation error (Tip 9), and no citation to support the arguments for Tip 2, 4, 6, 10, 11, 12.

If evidence from practice is presented, are all the underlying source data available to ensure full reproducibility?

Not applicable

Is the topic of the practical tips discussed accurately in the context of the current literature

Partly

Are all factual statements correct and adequately supported by citations?

Partly

Are the conclusions drawn balanced and justified on the basis of the presented arguments?

Partly

Are arguments sufficiently supported by evidence from the published literature and/or the authors’ practice?

Partly

Reviewer Expertise:

medical education, general medicine, peer learning

I confirm that I have read this submission and believe that I have an appropriate level of expertise to confirm that it is of an acceptable scientific standard, however I have significant reservations, as outlined above.

# Reviewer report: Reviewer response for version 1

**DOI:** 10.21956/mep.21344.r36229

**Published:** 2024-08-24

**Authors:** Stewart Mennin

**Affiliations:** 1 Human Systems Dyndamics Institute, Minneapolis, Minnesota, USA

Twelve tips for creating a medical education society is well presented and documented.  It assumes an already existing and interested group.  The article fits well within existing department or group of medical educators who are interested and ready to undertake this work. What I find missing in the article is how to get a group together so that they are cohesive and interested in peer teaching and faculty development. The most difficult part is getting a group together to collaborate, and this is not addressed in this article. However, assuming an existing group the article functions for a well and is very helpful. My experience is getting the group together is more difficult than doing the faculty development.

If evidence from practice is presented, are all the underlying source data available to ensure full reproducibility?

Partly

Is the topic of the practical tips discussed accurately in the context of the current literature

Yes

Are all factual statements correct and adequately supported by citations?

Yes

Are the conclusions drawn balanced and justified on the basis of the presented arguments?

Yes

Are arguments sufficiently supported by evidence from the published literature and/or the authors’ practice?

Yes

Reviewer Expertise:

My area of research and interest is in change in health professions organizations in all its levels from local individual through systemic and health systems themselves.

I confirm that I have read this submission and believe that I have an appropriate level of expertise to confirm that it is of an acceptable scientific standard.

# Reviewer report: Reviewer response for version 1

**DOI:** 10.21956/mep.21344.r35763

**Published:** 2024-02-29

**Authors:** Prattama Santoso Utomo

**Affiliations:** 1 Universitas Gadjah Mada, Yogyakarta, Indonesia

*The authors attempt to provide some tips for peer learning that are somewhat underestimated in medical education. Peer learning is important as an educational approach that could be beneficial for better outcomes of medical education. Some suggestions to improve the quality and consistency of the manuscript:*

*1) The authors might reconsider on the manuscript title. The current title "Twelve tips for creating a medical education society" is not well-aligned to the content of the tips, which focus on peer-learning and peer teaching activity suggestions. I think it will be better if the title is "Twelve tips for advancing peer teaching/education practices".*

*2) Introduction: the authors might want to ensure paragraphs are well-transitioned, to avoid a gap or jumping of ideas (particularly between the first and second paragraph).*

*3) The authors should consider supporting all tips with evidence/literature and/or actual experience from the authors.*

*4) Some tips were more conceptual rather than practical (i.e., Tip 4, Tip 10, Tip 11, Tip 12). More realistic examples and suggestions are needed for such tips.*

If evidence from practice is presented, are all the underlying source data available to ensure full reproducibility?

Not applicable

Is the topic of the practical tips discussed accurately in the context of the current literature

Partly

Are all factual statements correct and adequately supported by citations?

Partly

Are the conclusions drawn balanced and justified on the basis of the presented arguments?

Partly

Are arguments sufficiently supported by evidence from the published literature and/or the authors’ practice?

Partly

Reviewer Expertise:

medical education, general medicine, peer learning

I confirm that I have read this submission and believe that I have an appropriate level of expertise to confirm that it is of an acceptable scientific standard, however I have significant reservations, as outlined above.

# Response

**Published:** 2024-05-04

**Authors:** SOWMYA PRASANNA KUMAR MENON

**Affiliations:** MEDICINE, Keele University, Keele, England, UK

**Reviewer 3**  

**Reviewer comment 1:** The authors attempt to provide some tips for peer learning that are somewhat underestimated in medical education. Peer learning is important as an educational approach that could be beneficial for better outcomes of medical education. Some suggestions to improve the quality and consistency of the manuscript.  

**Response: **We thank the reviewer for their positive feedback. We provide a point-by-point response to each comment. We hope these improvements enable publication.

**Reviewer comment 2:** The authors might reconsider on the manuscript title. The current title "Twelve tips for creating a medical education society" is not well-aligned to the content of the tips, which focus on peer-learning and peer teaching activity suggestions. I think it will be better if the title is "Twelve tips for advancing peer teaching/education practices".  

**Response: **We thank the reviewer for their feedback. In the introduction, we outlined that the scope of this article is to provide guidance to medical students and enable them to create a medical education society. The authors Sowmya Prasanna Kumar Menon and Balamrit Singh Sokhal have fulfilled various committee roles (including chair) for their university medical education society. The foundation of this society is built from the philosophy of peer to peer teaching. Since joining, the society has grown to one of the largest at the university, and produced internationally recognised content, with presentation of results at conferences. Therefore this article aims to outline how peer-teaching can be optimised in the context of a medical education society, to facilitate peer collaboration and improve the next generation of clinicians and teachers. The manuscript title has been amended to reflect the inclusion of peer-teaching as suggested.  

***Under Title: “*** *Twelve tips for peer teaching by a medical education student society* *”*

***Under Introduction:*** “ *This article outlines practical and conceptual tips to develop, implement and expand a medical education society focussed on peer-teaching practices.”*  

**Reviewer comment 3:** Introduction: the authors might want to ensure paragraphs are well-transitioned, to avoid a gap or jumping of ideas (particularly between the first and second paragraph).  

**Response: **We thank the reviewer for their comment. We have amended the introduction to improve coherence between paragraphs.

***Under Introduction: “*** *A method to develop the clinician as an educator is through peer-teaching practices.”*  

**Reviewer comment 4:** The authors should consider supporting all tips with evidence/literature and/or actual experience from the authors.  

**Response: **We thank the reviewer for their feedback. We have amended the manuscript to include more citations where appropriate.  

**Reviewer comment 5:** Some tips were more conceptual rather than practical (i.e., Tip 4, Tip 10, Tip 11, Tip 12). More realistic examples and suggestions are needed for such tips. **Response: **We thank the reviewer for their feedback. The tips are based on what the authors who were heavily involved in their medical education society (Sowmya Prasanna Kumar Menon and Balamrit Singh Sokhal) experience or implemented during their tenure. There is little evidence to support these actions mainly due to the heterogeneity between educational practices and ever-changing requirements from students receiving sessions. Therefore, these tips should be treated as suggestions for readers wish to initiate a medical education society or further peer-teaching practices, rather than rules to follow. We believe an important aspect of developing a medical is to be flexible with the approach and adapt to the attendees the society is for. We hope this satisfactorily explains the reasons underlying the conceptual approach.

# Reviewer report: Reviewer response for version 1

**DOI:** 10.21956/mep.21344.r35760

**Published:** 2024-02-13

**Authors:** Masego B. Kebaetse

**Affiliations:** 1 University of Botswana, Gaborone, Botswana

We thank the authors for an interesting manuscript focused on providing learning support for students during medical training through peer teaching in medical education societies. We have the following comments:

1. We suggest that the authors engage with literature a bit more as part of the tips they are providing. Each tip, more or less, could be better supported with relevant literature.
2. Kindly remove the URL from tip # 12 and include it in the references where it is missing.
3. Tips #8 and #10, though labeled differently, seem to both speak about feedback. We suggest the authors find a way to differentiate the content of #10 from #8
4. We suggest that the authors review the tip headings and, where possible, revise them to match the content. For instance, I was not expecting Tip #7 to be just about managing the calendar and invitations based on the title.

If evidence from practice is presented, are all the underlying source data available to ensure full reproducibility?

Partly

Is the topic of the practical tips discussed accurately in the context of the current literature

Partly

Are all factual statements correct and adequately supported by citations?

Partly

Are the conclusions drawn balanced and justified on the basis of the presented arguments?

Yes

Are arguments sufficiently supported by evidence from the published literature and/or the authors’ practice?

No

Reviewer Expertise:

instructional design, teacher professional development, learning development/support

I confirm that I have read this submission and believe that I have an appropriate level of expertise to confirm that it is of an acceptable scientific standard, however I have significant reservations, as outlined above.

# Response

**Published:** 2024-05-04

**Authors:** SOWMYA PRASANNA KUMAR MENON

**Affiliations:** MEDICINE, Keele University, Keele, England, UK

**Reviewer 2** **Reviewer comment 1:** We thank the authors for an interesting manuscript focused on providing learning support for students during medical training through peer teaching in medical education societies. We have the following comments. **Response: **We thank the reviewer for their positive feedback. We provide a point-by-point response to each comment. We hope these improvements enable publication. **Reviewer comment 2:** We suggest that the authors engage with literature a bit more as part of the tips they are providing. Each tip, more or less, could be better supported with relevant literature. **Response: **We thank the reviewer for their feedback. We have amended the manuscript to be more clear so readers can reference the supporting literature when reading each tip. **Reviewer comment 3:** Kindly remove the URL from tip # 12 and include it in the references where it is missing. **Response: **We thank the reviewer for their comment. We have removed the URL from Tip 12 and included it in the references as suggested. **Reviewer comment 4:** Tips #8 and #10, though labelled differently, seem to both speak about feedback. We suggest the authors find a way to differentiate the content of #10 from #8 **Response: **We thank the reviewer for their feedback. To clarify, Tip 8 introduces the need for feedback and how to gather it, whereas Tip 10 proposes methods to act on that feedback with the aim to improve the society and develop in the direction attendees require. Some societies may find their attendees would like guest lectures, or different types of sessions (e.g. practical sessions, however other societies may find their attendees want specific sessions on different content (e.g. medical education research). **Reviewer comment 5:** We suggest that the authors review the tip headings and, where possible, revise them to match the content. For instance, I was not expecting Tip #7 to be just about managing the calendar and invitations based on the title. **Response: **We thank the reviewer for their feedback. We have amended the tip headings to better reflect their content. Including Tip 7. ***Under Tip 7: “*** *Advertise and leverage social media”*

# Reviewer report: Reviewer response for version 1

**DOI:** 10.21956/mep.21344.r35767

**Published:** 2024-02-13

**Authors:** Zakia Dimassi

**Affiliations:** 1 Khalifa University, Abu Dhabi, United Arab Emirates

Peer teaching is indeed a valuable and underutilized resource across the curriculum, and I find the concept behind this article very important and I would want to learn more about it. However, as I read through the 12 tips, I was underwhelmed as the tips came across as common sense, and add little to what medical educators already do as basic steps when introducing a new/novel intervention to the curriculum. 

 Tip 1: how about the perspective of the increasingly tech-savvy generation on how they learn and what grabs their attention?

 Tips 2 and 3: the eternal struggle is enhancing the content of the curriculum without overcrowding it. There is some attempt to address this here, although not sufficiently.

 Tip 4: faculty development is a pillar in MedEd and education in general, but how to make it more feasible and acceptable to busy clinicians?

 Tip 5: Indeed an excellent tip, but the supporting evidence is meager. Should draw from recent literature based on the COVID19 pandemic experiences with online platforms in MedEd.

 Tip 6: How to create valuable resources that can compete with platforms like Amboss, Osmosis efficiently and cost effectively? this is what I would have liked to see discussed

 Tip 7: there are few seminal articles on digital scholarship ref [1].  and online communities of practice (Gunwardena et al., 2016) that could have been nicely integrated it here to support the argument.

 Tip 8: how about the use of AI to enhance the usefulness and utility of feedback? Good-quality, curated, and timely feedback can become much easier to provide using tools like natural language processing

 Tip 9: I highly appreciate this tip. I would want to have it expanded on - certificates as part of CME.

 Tip 10: how to collect feedback data and how often? Feedback from whom? where to incorporate in the PDSA cycle or the Kern s steps?

 Tip 11: this is excellent as well, but again, how to fit in these sessions in an ever expanding curriculum?

 Tip 12: what about interprofessional and interdisciplinary per teaching to engrain this level of collaboration early on?

If evidence from practice is presented, are all the underlying source data available to ensure full reproducibility?

Not applicable

Is the topic of the practical tips discussed accurately in the context of the current literature

Partly

Are all factual statements correct and adequately supported by citations?

Partly

Are the conclusions drawn balanced and justified on the basis of the presented arguments?

Partly

Are arguments sufficiently supported by evidence from the published literature and/or the authors’ practice?

Partly

Reviewer Expertise:

curriculum development and evaluation, international medical graduates, team based learning, simulation-based education, patient safety in the medical curriculum

I confirm that I have read this submission and believe that I have an appropriate level of expertise to confirm that it is of an acceptable scientific standard, however I have significant reservations, as outlined above.

## References

[1] : Social Media and the 21st-Century Scholar: How You Can Harness Social Media to Amplify Your Career. *J Am Coll Radiol* .2018;15(1 Pt B) : 10.1016/j.jacr.2017.09.025 142-148 10.1016/j.jacr.2017.09.025 29154102

[2] : Analyzing social construction of knowledge Online by Employing Interaction Analysis, Learning Analytics, and Social Network Analysis. *UNM Digital Repository* .2016;

# Response

**Published:** 2024-05-04

**Authors:** SOWMYA PRASANNA KUMAR MENON

**Affiliations:** MEDICINE, Keele University, Keele, England, UK

**Reviewer 1**  

**Reviewer comment 1:** Peer teaching is indeed a valuable and underutilized resource across the curriculum, and I find the concept behind this article very important, and I would want to learn more about it. However, as I read through the 12 tips, I was underwhelmed as the tips came across as common sense and add little to what medical educators already do as basic steps when introducing a new/novel intervention to the curriculum.   

**Response: **We thank the reviewer for their comment and positive feedback on the manuscript. We provide a specific response to each of the reviewer’s comments below. This 12 tips article is intended to be a guide for medical students specifically who wish to initiate a medical education society in their medical school. We hope this guide gives them the confidence to incorporate these simple 12 tips in a peer-teaching format, without the need for formal medical education qualifications.

**Reviewer comment 2:** Tip 1: How about the perspective of the increasingly tech-savvy generation on how they learn and what grabs their attention?  

**Response: **We thank the reviewer for their feedback. We agree that students are now increasingly proficient in technology and that modern interventions should be incorporated to allow students to take control of their learning. Therefore, we have clarified that the initial ‘gap’ in students’ knowledge should be gathered in the form of digital surveys under tip 1. Furthermore, this tip focuses on how educators can identify topics to teach and not how to gather the audience, which is covered in the latter tips. Additionally, we have incorporated a variety of technological methods to advertise sessions and enhance learning which are not conventionally used.

***Under Tip 1:*** *“An effective digital survey is low resource, efficient, easy to complete and accessible through the distribution of QR codes.”*

**Reviewer comment 3:** Tips 2 and 3: The eternal struggle is enhancing the content of the curriculum without overcrowding it. There is some attempt to address this here, although not sufficiently.  

**Response: **We thank the reviewer for their comment. We agree that identifying content can be difficult in an institutional curriculum. However, we believe a student-led, medical education society’s aim should be to supplement the curriculum and faculty-led teaching. The aim is not to cover the entire curriculum. As stated in tip 1, societies should empower students to identify key topics the majority find difficult. Societies should function to provide extra time and resources dedicated to helping students understand these areas of weakness.

**Reviewer comment 4:** Tip 4: Faculty development is a pillar in MedEd and education in general, but how to make it more feasible and acceptable to busy clinicians?  

**Response: **We thank the reviewer for their comment. These tips are intended for students who would like to develop a medical education society in order to promote peer-to-peer teaching and mentorship. Therefore, although we agree clinicians are a valuable resource to medical education, they are beyond the scope of this article. We have amended tip 4 to reflect this. ***Under Tip 4:*** *These sessions should be developed in collaboration with faculty lecturers to incorporate their extensive experience and advice.*

**Reviewer comment 5:** Tip 5: Indeed an excellent tip, but the supporting evidence is meager. Should draw from recent literature based on the COVID19 pandemic experiences with online platforms in MedEd.  

**Response: **We thank the reviewer for their feedback. The evidence we have referenced is contemporary published in 2020 and 2021 and derived from the experience of educators during the pandemic.

***Under References section:*** *5 **. *** Kumari S, Gautam H, Nityadarshini N, et al.: Online classes versus traditional classes? Comparison during COVID-19. *J Educ Health Promot.* 2021;10:457. 35233404 8826787 *6. Mukhtar K, Javed K, Arooj M, et al.: Advantages, Limitations and Recommendations for online learning during COVID-19 pandemic era. Pak J Med Sci. 2020;36(COVID19-S4):S27–S31. 32582310 10.12669/pjms.36.COVID19-S4.2785 7306967*

**Reviewer comment 6:** Tip 6: How to create valuable resources that can compete with platforms like Amboss, Osmosis efficiently and cost effectively? this is what I would have liked to see discussed.  

**Response: **We thank the reviewer for their comment. The scope of this article is to cover creating a medical education society purely for students at the local educational institution. The aim is not to compete with international, established platforms, but rather to provide a personalized experience to students based on their university-specific curriculum. Conversely, platforms that have helped tutors may be recommended to students during these sessions as part of further learning.

**Reviewer comment 7: ** Tip 7: There are few seminal articles on digital scholarship ref [1].  and online communities of practice (Gunwardena et al., 2016) that could have been nicely integrated it here to support the argument.  

**Response: **We thank the reviewer for their feedback and are pleased that the reviewer found the references useful. We have reviewed the suggested reference by Gunwardena et al, and believe this does not add to the reference we have cited. Therefore, we have made the decision to not incorporate this reference in the manuscript.

**Reviewer comment 8:** Tip 8: How about the use of AI to enhance the usefulness and utility of feedback? Good quality, curated, and timely feedback can become much easier to provide using tools like natural language processing.  

**Response: **We thank the reviewer for their comment. The use of artificial intelligence is an important topic. Research about the use of artificial intelligence to gather feedback is lacking. In addition, the authors did not extensively utilise artificial intelligence during their peer-teaching practices. Therefore, whilst we agree that the use of artificial intelligence could be beneficial, it should be used adjunct to conventional methods. We have stated this under Tip 8.  

***Under Tip 8:*** *Artifical intelligence (AI) tools could also be used to enhance the efficiency of the feedback process and present data comprehensively.* *However, these tools must be used with caution as we are still in the early stages of implementation of AI.*

**Reviewer comment 9:** Tip 9: I highly appreciate this tip. I would want to have it expanded on - certificates as part of CME.  

**Response: **We thank the reviewer for their feedback. This article covers peer-led medical student teaching under a local society. Therefore, this teaching may not qualify for CME.

**Reviewer comment 10:** Tip 10: How to collect feedback data and how often? Feedback from whom? where to incorporate in the PDSA cycle or the Kern s steps?  

**Response: **We thank the reviewer for their comment. We believe the PDSA cycle is fundamental knowledge for educators. We have incorporated this feedback under Tip 10. ***Under Tip 10:*** *Feedback is only useful if acted upon in accordance with the plan-do-study-act cycle (PDSA). Feedback should be gathered immediately after each session and incorporated at the earliest convenience when planning the next session.*  

**Reviewer comment 11:** Tip 11: This is excellent as well, but again, how to fit in these sessions in an ever expanding curriculum?  

**Response: **We thank the reviewer for their comment. As the society is student-led and peer-taught, sessions will be planned in real-time to keep up with an ever-changing curriculum. As mentioned, content is planned in conjunction with feedback from prior sessions. Commonly sessions are held out-of-hours as a supplement to faculty-led teaching, and not a replacement. Therefore, the session learning objectives will be in line with the curriculum and versatile according to changes.

**Reviewer comment 12:** Tip 12: What about interprofessional and interdisciplinary per teaching to engrain this level of collaboration early on?  

**Response: **We thank the reviewer for their feedback. Interprofessional education is important and can provide valuable insight into the wider healthcare team. We have implemented this under tip 12.  

***Under Tip 12:*** *This can be extended to the wider health professions community. Interprofessional collaboration in sessions such as mock OSCEs through simulation can provide invaluable insight into other healthcare roles such as pharmacy, nursing and physiotherapy.*
